# Inhibition of oxidative phosphorylation for enhancing citric acid production by *Aspergillus niger*

**DOI:** 10.1186/s12934-015-0190-z

**Published:** 2015-01-16

**Authors:** Lu Wang, Jianhua Zhang, Zhanglei Cao, Yajun Wang, Qiang Gao, Jian Zhang, Depei Wang

**Affiliations:** Key Laboratory of Industrial Fermentation Microbiology, Ministry of Education, Tianjin, 300457 P. R. China; College of Biotechnology, Tianjin University of Science & Technology, Tianjin, 300457 P. R. China; Tianjin Key Laboratory of Industrial Microbiology, Tianjin, 300457 P. R. China

**Keywords:** Citric acid, *Aspergillus niger*, Oxidative phosphorylation inhibitor, Uncoupler of oxidative phosphorylation, Energy metabolism

## Abstract

**Background:**

The spore germination rate and growth characteristics were compared between the citric acid high-yield strain *Aspergillus niger* CGMCC 5751 and *A. niger* ATCC 1015 in media containing antimycin A or DNP. We inferred that differences in citric acid yield might be due to differences in energy metabolism between these strains. To explore the impact of energy metabolism on citric acid production, the changes in intracellular ATP, NADH and NADH/NAD^+^ were measured at various fermentation stages. In addition, the effects of antimycin A or DNP on energy metabolism and citric acid production was investigated by CGMCC 5751.

**Results:**

By comparing the spore germination rate and the extent of growth on PDA plates containing antimycin A or DNP, CGMCC 5751 was shown to be more sensitive to antimycin A than ATCC 1015. The substrate-level phosphorylation of CGMCC 5751 was greater than that of ATCC 1015 on PDA plates with DNP. DNP at tested concentrations had no apparent effect on the growth of CGMCC 5751. There were no apparent effects on the mycelial morphology, the growth of mycelial pellets or the dry cell mass when 0.2 mg L^-1^ antimycin A or 0.1 mg L^-1^ DNP was added to medium at the 24-h time point. The concentrations of intracellular ATP, NADH and NADH/NAD^+^ of CGMCC 5751 were notably lower than those of ATCC 1015 at several fermentation stages. Moreover, at 96 h of fermentation, the citric acid production of CGMCC 5751 reached up to 151.67 g L^-1^ and 135.78 g L^-1^ by adding 0.2 mg L^-1^ antimycin A or 0.1 mg L^-1^ DNP, respectively, at the 24-h time point of fermentation. Thus, the citric acid production of CGMCC 5751 was increased by 19.89% and 7.32%, respectively.

**Conclusions:**

The concentrations of intracellular ATP, NADH and NADH/NAD^+^ of the citric acid high-yield strain CGMCC 5751 were notably lower than those of ATCC 1015. The excessive ATP has a strong inhibitory effect on citric acid accumulation by *A. niger*. Increasing NADH oxidation and appropriately reducing the concentration of intracellular ATP can accelerate glycolysis and the TCA cycle to enhance citric acid yield.

**Electronic supplementary material:**

The online version of this article (doi:10.1186/s12934-015-0190-z) contains supplementary material, which is available to authorized users.

## Background

Citric acid is widely used in the pharmaceutical, cosmetic, food and chemical industries and recently has been produced by an industrial-scale process of fermentation using the filamentous fungus *Aspergillus niger* as a microbial cell factory [1]. Citric acid production by fermentation is the most economical and widely used way of obtaining this product, and more than 90% of the world’s citric acid is currently produced in this way [2]. Zahorsky (1913) first discovered that citric acid can be produced by *A. niger* [3]. Currie (1917) laid the preliminary theoretical foundation of citric acid production by fermentation [4]. The metabolism in *A. niger* during this process has been reviewed recently [5-7], and crucial development events in primary metabolism have been described to increase our understanding of the process. Because the concentration of extracellular citric acid accumulated by *A. niger* reaches 150–200 g L^−1^ under suitable conditions, this “metabolic overflow” has attracted some biochemical interest and has been a model example for investigating why cells overproduce organic acids from the tricarboxylic acid (TCA) cycle. The glucose uptake rate has been identified by Torres [8,9] as an important factor for the rate of citric acid production. They apply mathematical modelling to predict that the glycolytic reactions of *A. niger* are limited by the supply of the initial substrate and the removal of the final product.

The glycolytic pathway is one of the most thoroughly studied biochemical pathways. Hexokinase, phosphofructokinase and pyruvate kinase are rate-limiting enzymes for the glycolytic pathway in most microorganisms [10]. Initially, to promote glycolytic metabolism, the inhibitors of these three rate-limiting enzymes must be removed. However, some studies revealed that single or joint over-expression of the enzymes could not significantly improve the rate of glycolytic flux in yeast [6] or bacteria [11].

Analysis of metabolic control indicated that glycolytic flux was influenced by intracellular ATP concentrations. High concentrations of ATP restrained phosphofructokinase activity, but ADP activated the activity of both phosphofructokinase and hexokinase [12]. Therefore, the reduction of ATP levels increased glycolytic flux substantially [13,14]. Conversely, some scientists have studied the effect of coenzyme NAD^+^ on the regulation of glycolysis. Vemuri [15] overexpressed NADH oxidase and reduced intracellular NADH/NAD^+^ in *Saccharomyces cerevisiae*, and as a result, the glycolytic flux increased. Analogously, Neves [16] studied the relationship between NAD^+^ and the glycolytic pathway using isotope tracer technology in *Lactococcus lactis* and discovered that reducing the level of NAD^+^ or blocking the oxidation of NADH would lead to an apparent decrease in glycolytic flux. In summary, to increase glycolytic flux and promote citric acid output, an effective strategy is to decrease ATP concentration and simultaneously oxidize NADH to NAD^+^. Oxidative phosphorylation and substrate-level phosphorylation are the two aerobic ATP biosynthetic pathways in the cell [17,18]. Oxidative phosphorylation is the main pathway for ATP synthesis and hence plays an important role in maintaining ATP levels in the cell. Substrate-level phosphorylation is a supplementary pathway for ATP synthesis, which directly generates ATP and NADH without the electron transport chain. Therefore, blocking or restraining oxidative phosphorylation can effectively decrease ATP concentrations in the cell.

To enhance the yield of citric acid by *A. niger* in submerged fermentation, various factors relating to cell cultivation have been considered. Surprisingly, some antibiotics and inhibitors have been positively applied to promote the production of citric acid. For example, as an inhibitor of oxidative phosphorylation, antimycin A can inhibit succinate-cytochrome c reductase in the electron transfer chain to block NADH oxidation and ATP synthesis. Liu et al. [19] added antimycin A to the broth of *Torulopsis glabrata* CCTCC M202019, which significantly decreased the intracellular ATP concentration (27.7%) and apparently increased the rate of glucose consumption (240%). DNP, a typical uncoupler of oxidative phosphorylation [20], separates the coupled process of NADH oxidation and ATP synthesis into two independent processes. DNP has no influence on the electron transfer chain but blocks ATP synthesis. In addition, by adding DNP to the medium, the process that synthesizes ATP through oxidative phosphorylation was inhibited so that accumulated ADP promotes substrate-level phosphorylation. Dietzler et al. [21] added DNP to the culture broth of *Escherichia coli* and found that intracellular ATP levels decreased and glucose metabolism accelerated.

In this study, for both the citric acid high-yield strain CGMCC 5751 and the standard strain ATCC 1015, differences in energy metabolism were demonstrated by observing the spore germination rate and the extent of growth on PDA plates and measuring the concentration of intracellular ATP, NADH and NADH/NAD^+^. However, the main aim of this study was to enhance the citric acid production by regulating the concentrations of intracellular ATP, NADH and NADH/NAD^+^ [19-21]. Because there is a strong relationship between the early mycelial pellet morphology and the final yield of citric acid, the influence of intracellular ATP, NADH levels and NADH/NAD^+^ on the growth and citric acid fermentation of *A. niger* were investigated by adding antimycin A or DNP to the media, and the differences in energy metabolism relative to *A. niger* CGMCC 5751 were studied. Furthermore, the effects of antimycin A or DNP were evaluated for their effects on citric acid yield by *A. niger* CGMCC 5751.

## Results and discussion

### Effect of antimycin A on spore germination of *A. niger*

**Figure 1:**
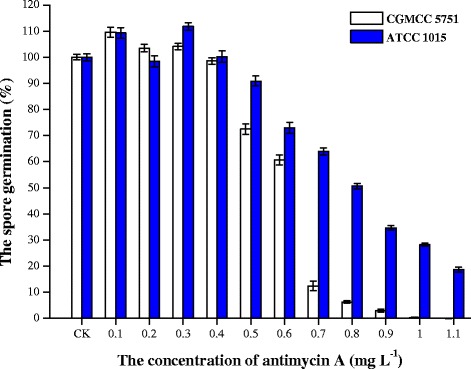
**The effect of antimycin A concentration on spore germination rate of** ***A. niger*** **CGMCC 5751 and ATCC 1015.** ***A. niger*** **cultivated on PDA plate containing 0.1–1.1 mg L** ^**-1**^ **antimycin A.** Data are average values and standard deviations of triplicate.

The results showed that antimycin A has an inhibitory effect on the spore germination of *A. niger* (Figure 1). The spore germination of the standard strain ATCC 1015 was reduced slowly, while that of the citric acid high-yielding strain CGMCC 5751 decreased rapidly. When the concentration of antimycin A in PDA plates was less than 0.7 mg L^−1^, the spore germination rate was over 60% for both strains, but by adding 0.7 mg L^−1^ antimycin A, the spore germination rate of CGMCC 5751 was only 12.39%, meanwhile that of ATCC 1015 was 63.87%; the spores of CGMCC 5751 could hardly germinate when antimycin A was present at a concentration greater than 0.9 mg L^−1^ (Figure 1). Consequently, CGMCC 5751 was more sensitive to antimycin A than was ATCC 1015.

**Figure 2:**
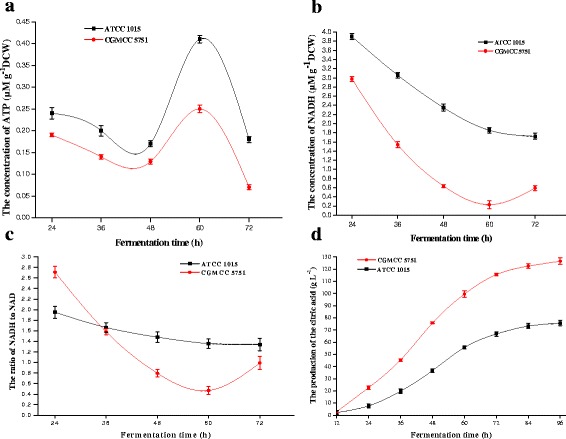
**The concentrations of intracellular ATP (a), NADH (b), NADH/NAD (c) and the production of citric acid (d) of CGMCC 5751 and ATCC 1015 at different time points in liquefied corn medium at 350 r min** ^**-1**^ **and 35°C for 72 h in 30-L fermentors.** Data are average values and standard deviations of triplicate experiments.

With the highest concentration of antimycin A, the two tested strains produce so little energy by oxidative phosphorylation that the spore germination rate was reduced markedly; however, if there are other metabolic pathways that oxidize NADH or produce ATP, the spore germination rate should decline more slowly. Compared with ATCC 1015, the germination rate of CGMCC 5751 was significantly lower, probably because of the weakness of the metabolic energy pathways for ATP synthesis in the spore germination phase after adding antimycin A. In contrast, ATCC 1015 has relatively stronger metabolic energy pathways. These results will also be tested below (Figure 2abc).

### The effect of antimycin A on the growth of *A. niger*

For both CGMCC 5751 and ATCC 1015, the experimental group always grew slower than the control. The reason for this observation is that antimycin A has an inhibitory effect on ATP synthesis via oxidative phosphorylation, and colonies grow slowly due to the lack of ATP (data not shown). Moreover, by comparing the control groups with the experimental groups, CGMCC 5751 exhibited almost a 24-h slower growth than ATCC 1015, and the colony diameter of CGMCC 5751 was also markedly smaller (data not shown). We inferred that ATCC 1015 has a strong metabolic energy pathway to synthesize ATP for its growth, while CGMCC 5751 is weaker in this pathway, and these results were consistent with previous analysis. This conclusion is also demonstrated by the results of the following experiments (Figure 2abc).

To further define the difference in metabolic energy pathways between CGMCC 5751 and ATCC 1015, the effect of DNP on *A. niger* was evaluated in the following experiments.

### The effect of DNP on spore germination of *A. niger*

**Figure 3:**
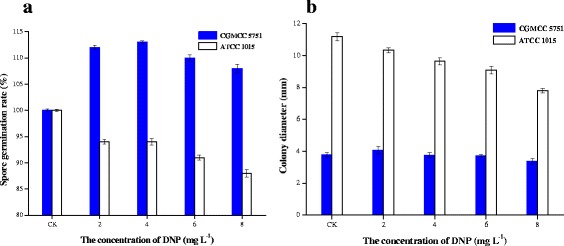
**Citric acid output of CGMCC 5751 in liquefied corn medium. a**. different adding time point of 0.2 mg L^-1^ antimycin A. **b**. adding different antimycin A concentrations at the 24-h time point. The amount of 1×10^5^ spores mL^-1^ of *A. niger* cultivated in liquefied corn medium at 350 r min^-1^ and 35°C for 72 h in shake flasks. Data are average values and standard deviations of triplicate experiments.

On PDA medium, the two strains had similar germination rates, but the germination rate of ATCC 1015 with added DNP decreased compared to its corresponding control (without DNP). In contrast, the germination of CGMCC 5751 in the presence of DNP was higher than that of its corresponding control (Figure 3a). For ATCC 1015, DNP led to a sharp decrease in the amount of energy produced by oxidative phosphorylation; therefore, ATCC 1015 only relies on substrate-level phosphorylation to generate ATP. The decreased spore germination rate indicated that the total ATP synthesis of ATCC 1015 with DNP was lower than that of the control on PDA medium. However, for CGMCC 5751, its spore germination rate increased to 110% on PDA medium with DNP, which suggested that total ATP synthesis is higher than the control (without DNP) in the growth phase. When the cyanide-sensitive respiratory chain was gradually inactivated by adding DNP, ATP synthesis was weak due to inhibition of oxidative phosphorylation. Nevertheless, *A. niger* has a salicylhydroxamic acid-sensitive respiration chain that oxidizes NADH to NAD^+^. Additionally, DNP causes an immense accumulation of ADP and promotes ATP synthesis through substrate-level phosphorylation. Because there is no additional metabolic pathway to synthesize ATP, ATP synthesis of CGMCC 5751 is wholly dependent on substrate-level phosphorylation. Here, the spore germination rate under DNP stress was higher than that of the control, indicating that total ATP synthesis of CGMCC 5751 with DNP was higher than that of the control on PDA medium. In brief, the results demonstrate that the substrate-level phosphorylation of the high-yield strain CGMCC 5751 was stronger than that of standard strain ATCC 1015.

### The effect of DNP on the growth of *A. niger*

With the increase in DNP concentration, the colony diameter of ATCC 1015 gradually decreased because the pathway that synthesizes ATP by oxidative phosphorylation was completely inhibited. Superabundant ATP may inhibit the salicylhydroxamic acid-sensitive respiratory chain to reduce the growth rate of ATCC 1015.

On DNP-containing PDA medium, the diameter of CGMCC 5751 colonies did not change compared with the control group without DNP (Figure 3b). The reason for this observation may be that DNP promotes ATP synthesis by substrate-level phosphorylation but inhibits the ATP synthesis by oxidative phosphorylation in CGMCC 5751. Thus, the growth rate of CGMCC 5751 remained unchanged.

Based on the above results, the metabolic energy performance during the growth stage was preliminarily outlined for CGMCC 5751 and ATCC 1015 on PDA plates by adding antimycin A or DNP. We infer that DNP has a positive effect on the substrate-level phosphorylation pathway of CGMCC 5751 and ensures its vigorous growth compared with the control (without DNP). Compared with ATCC 1015, CGMCC 5751 may lack an energy metabolic pathway to synthesize adequate ATP in the growth stage, which results in ATP deficiency and a slower growth rate. Consequently, its colony diameter was only approximately one-third that of ATCC 1015 (Figure 3b).

### The effect of antimycin A at different time points on citric acid fermentation by CGMCC 5751

**Figure 4:**
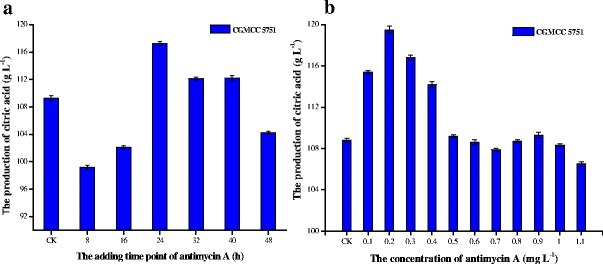
**Citric acid output of CGMCC 5751 in liquefied corn medium. a**. different adding time point of 0.1 mg L^-1^ DNP **b**. adding different DNP concentrations at the 24-h time point. The amount of 1×10^5^ spore mL^-1^ of *A. niger* was cultivated in liquefied corn medium at 350 r min^-1^ and 35°C for 72 h in shake flasks. Data are average values and standard deviations of triplicate experiments.

The citric acid fermentation process of *A. niger* can be divided into the growth stage (0–24 h) and the citric acid production stage (24–72 h). We found that citric acid output reached a maximum value upon adding 0.2 mg L^−1^ antimycin A at the 24-h time point of fermentation (Figure 4a). Adding antimycin A just before the citric acid production stage improved citric acid output (P = 0.000). If added at the growth stage, antimycin A inhibits growth and results in decreased citric acid production; on the contrary, because ATP is in relatively low demand during the citric acid production stage, adding antimycin A had a small or negative effect on citric acid output (Figure 4a).

### The effect of antimycin A concentration on citric acid fermentation by CGMCC 5751

With increasing concentrations of antimycin A, citric acid output was initially enhanced and subsequently decreased. When antimycin A was added to 0.2 mg L^−1^ at the 24-h time point, the output increased to a maximum level (Figure 4b). Because small amounts of antimycin A inhibit ATP synthesis and remove its feedback inhibition on phosphofructokinase, glycolysis is improved to enhance citric acid output. The cyanide-sensitive respiratory chain of CGMCC 5751 is excessively weakened and even blocked by increasing concentrations of antimycin A, which could be demonstrated by the change in intracellular ATP and NADH concentrations during fermentation. Cell growth is severely affected at the beginning, and citric acid output is affected later. Thus, the higher concentration of antimycin A results in lower citric acid production.

### The effect of DNP addition time on citric acid fermentation by CGMCC 5751

**Figure 5:**
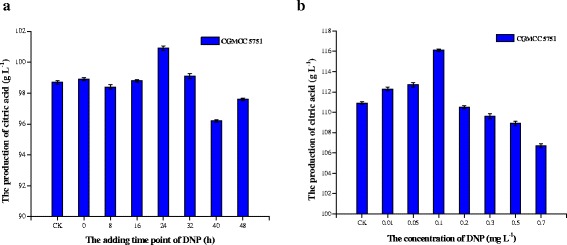
**Effect of 0.2 mg L** ^**-1**^ **antimycin A or 0.1 mg L** ^**-1**^ **DNP on the growth characteristics of CGMCC 5751 in liquefied corn medium at 350 r min** ^**-1**^ **and 35°C for 72 h in 30-L fermentors. a**. the mycelial pellets morphology **b**. the diameter of mycelial pellets **c**. the dry cell mass. Data are average values and standard deviations of triplicate experiments.

When 0.1 mg L^−1^ DNP was added to liquefied corn medium at different time points, the citric acid output peaked upon addition of DNP at the 24-h time point (Figure 5a). In the above experiments, it can be seen that CGMCC 5751 entered the peak of citric acid production after 24 h. When added at the 24-h time point, DNP not only had no visible effect on the morphology of mycelia pellets (P = 0.813) but also provided the appropriate ATP concentrations by substrate-level phosphorylation for citric acid production. Accordingly, the addition of 0.1 mg L^−1^ to liquefied corn medium at the 24-h time point is the most efficient means to regulate ATP synthesis and citric acid production.

### The effect of DNP concentration on citric acid fermentation by CGMCC 5751

With increasing concentrations of DNP, citric acid output increased initially and then decreased. When 0.1 mg L^−1^ DNP was added to liquefied corn medium at the 24-h time point, the citric acid output achieved a maximum (Figure 5b). When DNP was less than 0.1 mg L^−1^, we infer that DNP stimulated the glycolytic pathway and heightened pyruvate levels to promote citric acid production. With increasing concentrations of DNP, ATP synthesis was drastically reduced through oxidative phosphorylation, which results in a deficiency of ATP. Because ATP is required in the process of citric acid production, the citric acid output decreased.

### The effect of antimycin A or DNP on the growth of CGMCC 5751 in liquefied corn medium

**Figure 6:**
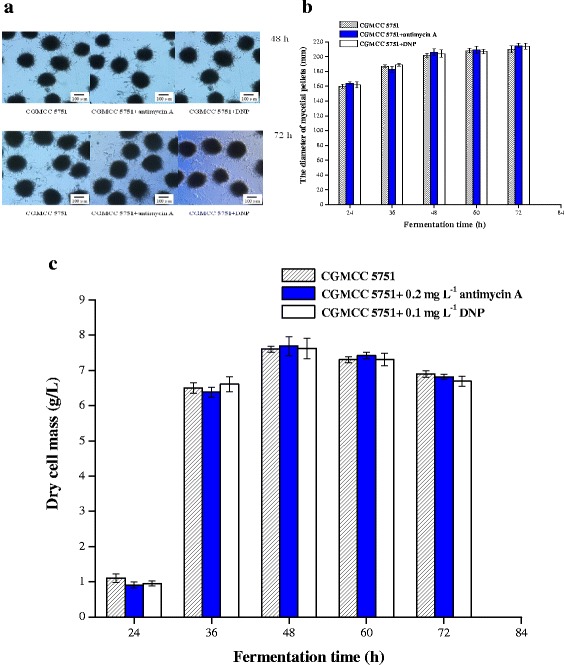
**Effect of 0.2 mg L** ^**-1**^ **antimycin A or 0.1 mg L** ^**-1**^ **DNP on the morphology (a)and diameter of mycelial pellets (b) and dry cell mass of CGMCC 5751 (c) in liquefied corn medium at 350 r min** ^**-1**^ **and 35°C for 72 h in 30-L fermentors.** Data are average values and standard deviations of triplicate experiments.

The results illustrated no apparent effect on the mycelial morphology, the growth of mycelial pellets or the dry cell mass when 0.2 mg L^−1^ antimycin A or 0.1 mg L^−1^ DNP was added to liquefied corn medium at the 24-h time point of fermentation (Figure 6abc). The results were consistent with the mycelial growth on PDA plates containing 0.2 mg L^−1^ antimycin A or 0.1 mg L^−1^ DNP; there were also no significant changes in spore germination and colony diameter (Figures 1 and 3ab).

### Measurement of intracellular ATP, NADH and NADH/NAD^+^ levels in citric acid fermentation

From the perspective of ATP suppression, the mechanism of improving citric acid production is explored by measuring the variations in ATP, NADH and NADH/NAD^+^ concentrations at different fermentation stages for both strains. At the 24–48 h stage, a large amount of energy was needed to transport intracellular nutrients, to biosynthesize intermediates and to support the fast growth of mycelia; meanwhile, the rates of glycolysis and the TCA cycle were relatively slow in this phase. Consequently, the formation rates of ATP and NADH were less than their consumption rates during the growth stage, and the intracellular ATP, NADH and NADH/NAD^+^ levels were reduced for both strains (Figure 2abc). During the 48–72 h stage, the glycolysis and TCA cycles were accelerated to produce large amounts of ATP. The cyanide-sensitive and salicylhydroxamic acid-sensitive respiration chains are constitutive in *A. niger* [22-24], which is different than what is reported in *Neurospora crassa 56* [25,26] and *Hansenula anomala 7* [27,28]. For CGMCC 5751, ATP synthesis by the cyanide-sensitive respiration chain was blocked in the previous experiments [29]. When cyanide-sensitive respiration was inactive, the salicylhydroxamic acid-sensitive pathway was activated to compensate [30,31]. However, the latter pathway produces less ATP than the cytochrome pathway [29]. Furthermore, intracellular NADH can continue to be oxidized, so intracellular ATP concentrations were lower than those in ATCC 1015 (Figure 2a). However, for ATCC 1015, this pathway was inactive because of the cyanide-sensitive respiration chain, suggesting that intracellular NADH and NADH/NAD^+^ levels were higher than those of CGMCC 5751 (Figure 2bc). Because the transcription level of the alternative oxidase that catalysed the salicylhydroxamic acid-sensitive respiration chain is weak at the 60–72 h stage in *A. niger* WU-2223L [29], NADH could hardly be oxidized by the alternative oxidase. As a result, the glycolysis pathway as well as the TCA cycle is strongly inhibited by the excessive ATP [1]. Therefore, intracellular ATP levels decreased for the two strains, but the NADH level was slightly elevated in CGMCC 5751. Finally, CGMCC 5751 produced much higher citric acid levels than ATCC 1015 (Figure 2d). Hence, citric acid production has a strong relationship with the higher concentrations of intracellular ATP and NADH.

**Figure 7:**
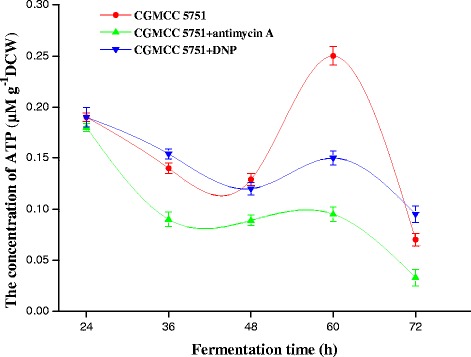
**The intracellular ATP concentration of CGMCC 5751 at different time points in liquefied corn medium in 30-L fermentors.** The amount of 0.2 mg L^-1^ antimycin A or 0.1 mg L^-1^ DNP was added to liquefied corn medium at the 24-h time point of fermentation. Data are average values and standard deviations of triplicate experiments.

**Figure 8:**
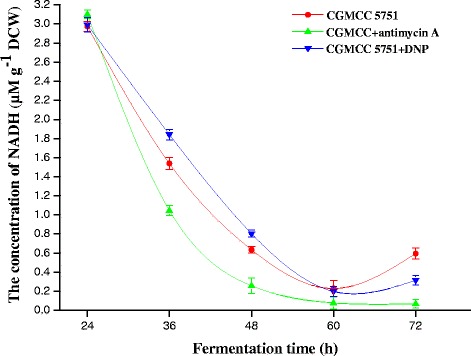
**The intracellular NADH concentration of CGMCC 5751 at different time points in liquefied corn medium in 30-L fermentors.** The amount of 0.2 mg L^-1^ antimycin A or 0.1 mg L^-1^ DNP was added to liquefied corn medium at the 24-h time point of fermentation. Data are average values and standard deviations of triplicate experiments.

**Figure 9:**
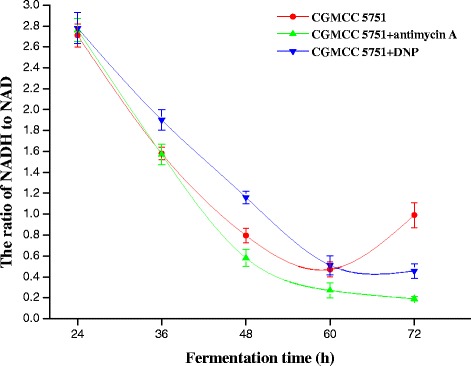
**The NADH/NAD** ^**+**^ **ratio of CGMCC 5751 at different time points in liquefied corn medium in 30-L fermentors.** The amount of 0.2 mg L^-1^ antimycin A or 0.1 mg L^-1^ DNP was added to liquefied corn medium at the 24-h time point of fermentation. Data are average values and standard deviations of triplicate experiments.

**Figure 10:**
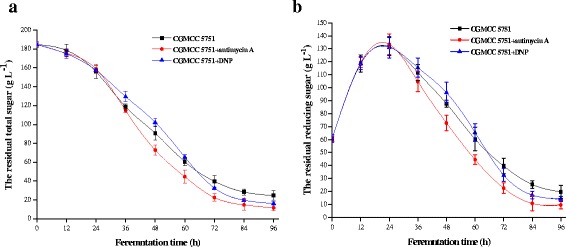
**The contents of residual total sugar (a) and the residual reducing sugar (b) in fermentation broth at different time points in liquefied corn medium in 30-L fermentors.** The amount of 0.2 mg L^-1^ antimycin A or 0.1 mg L^-1^ DNP was added to liquefied corn medium at the 24 h time point of fermentation. Data are average values and standard deviations of triplicate experiments.

**Figure 11:**
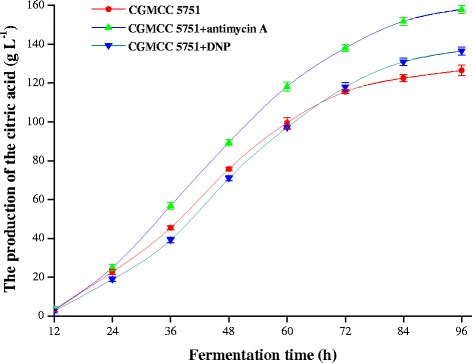
**The production of citric acid of CGMCC 5751 at different time points in liquefied corn medium in 30-L fermentors.** The amount of 0.2 mg L^-1^ antimycin A or 0.1 mg L^-1^ DNP was added to liquefied corn medium at the 24 h time point of fermentation. Data are average values and standard deviations of triplicate experiments.

For CGMCC 5751, the intracellular ATP levels not only ensure that there is no inhibition for various rate-limiting enzymes but also appropriately promote the glycolysis and TCA cycles. However, when 0.2 mg L^−1^ antimycin A was added to liquefied corn medium at the 24-h time point of fermentation, the intracellular ATP concentration was substantially suppressed. At the 24–72 h stage, all intracellular ATP concentrations were lower than the control and remained stable (Figure 7), as did the intracellular NADH concentration and NADH/NAD^+^ (Figures 8 and 9). In this way, the inhibition was released to accelerate glycolysis and the TCA cycle, and the consumption rate of total sugar or reducing sugar was accelerated (Figure 10ab). Therefore, the rate of citric acid production was very fast, and the yield was very high (Figure 11). When added to liquefied corn medium at the 24-h time point of fermentation, at the stage of 24–60 h, 0.1 mg L^−1^ DNP inhibited the generation of ATP formed by oxidative phosphorylation. The intracellular ATP level was lower than the control without DNP treatment, as was the consumption rate of total sugar and reduced sugar (Figure 10ab). These results confirm that glycolysis and the rate of citric acid production were suppressed compared to the control (Figure 11). Nevertheless, NADH oxidation still continued such that the intracellular NADH concentration and NADH/NAD^+^ were higher than the no DNP control. In addition, NADH was also oxidized through the salicylhydroxamic acid-sensitive respiration chain, and substrate-level phosphorylation continued. As a consequence, intracellular ATP levels were higher than those found in the group treated with antimycin A. At the 60–72 h stage, intracellular concentrations of ATP, NADH and NADH/NAD^+^ in the DNP-adding group were lower than the control (Figures 7, 8 and 9); whereas the rate of total sugar consumption and reduced sugar was higher than the control (Figure 10ab). The inhibition of both ATP and NADH weakened the glycolysis and TCA cycle; thence, the citric acid production was superior to the control (Figure 11), but the intracellular concentrations of ATP, NADH and NADH/NAD^+^ were higher than those seen in the group treated with antimycin. The result is that the citric acid production was lower than that of the latter group. Finally, at the end of fermentation, the citric acid production of CGMCC 5751 reached up to 151.67 g L^−1^ and 135.78 g L^−1^ by adding 0.2 mg L^−1^ antimycin A or 0.1 mg L^−1^ DNP, respectively, at the 24-h time point of fermentation; however, the control group citric acid levels were only 126.51 g L^−1^. Therefore, at the end of fermentation, the citric acid production was significant enhanced by adding 0.2 mg L^−1^ antimycin A (P = 0.000) or 0.1 mg L^−1^ DNP (P = 0.002). Accordingly, it can be concluded that, first, the excess ATP plays a strong regulatory role in the salicylhydroxamic acid-sensitive respiration chain in *A. niger*; second, the excess ATP has a strong inhibitory effect on citric acid accumulation; and, third, it can accelerate glycolysis and the TCA cycle by strengthening the ability of NADH oxidation and appropriately reducing the concentration of intracellular ATP.

## Conclusions

In the present study, antimycin A or DNP causes a difference in spore germination and growth between the citric acid high-yield strain CGMCC 5751 and the standard strain ATCC 1015. Comparing the growth extent of the two strains on PDA media containing antimycin A or DNP revealed that antimycin A significantly inhibited the spore germination of both strains, but CGMCC 5751 was more sensitive to antimycin A than was ATCC 1015. DNP had a positive impact on substrate-level phosphorylation for CGMCC 5751 that slightly increased the spore germination rate of CGMCC 5751, but the growth rate was not affected in the presence of DNP (P = 0.337) and was inhibited for ATCC 1015. However, there exist ATP synthesis deficiencies or nutritional deficiencies in CGMCC 5751, which result in slow growth compared with ATCC 1015, such that it could not grow normally on the PDA medium with antimycin A concentrations above 0.7 mg L^−1^. Moreover, the concentrations of intracellular ATP, NADH and NADH/NAD^+^ of CGMCC 5751 were notably lower than those of ATCC 1015 at various fermentation stages.

The mechanism for improving citric acid production was explored by adding energy metabolism inhibitors to the fermentation broth for strain CGMCC 5751. When 0.2 mg L^−1^ antimycin A or 0.1 mg L^−1^ DNP was added to liquefied corn medium at the 24-h time point of fermentation, the intracellular ATP concentration was substantially suppressed, but the NADH/NAD^+^ of the experimental group was lower than the control group during citric acid production. From the perspective of energy suppression, our experiments enhanced our understanding of the mechanism of citric acid production in the high-yield strain and the relationship between carbon-centred metabolism and energy metabolism in *A. niger*. It can be concluded that excess ATP has a strong inhibitory effect on citric acid accumulation and that strengthening NADH oxidation and reducing the concentration of intracellular ATP can accelerate glycolysis (Figure 10ab) and the TCA cycle to enhance citric acid yield (Figure 11). It is expected that more evidence will be found to clarify the regulation mechanism of ATP and NADH levels for central metabolic pathways. Moreover, increasing the consumption rate of the substrate by weakening the energy metabolism pathways was appropriate for obtaining a higher yield of citric acid. Such an approach may be generally applicable to improve yields from various industrial fermentation strains.

Furthermore, the existence of the *aox* gene encoding an alternative oxidase in the salicylhydroxamic acid-sensitive respiration chain [32] was confirmed in the citric acid-producer *A. niger* CGMCC 5751. The *aox* genes and their amino acid sequences have been aligned for both CGMCC 5751 and ATCC 1015. Our future work of disruption or overexpression of *aox* in the citric acid producer CGMCC 5751 will be performed to further explore the effects of energy metabolism by the salicylhydroxamic acid-sensitive respiration chain on citric acid production.

## Materials and methods

### Microorganisms

*Aspergillus niger* CGMCC 5751, a hyper-producer of citric acid fermentation, was obtained from Tianjin Key Laboratory of Industrial Microbiology, Tianjin, China. The standard strain *A. niger* ATCC 1015 was purchased from the American Type Culture Collection (ATCC, Rockville, MD, USA).

### Media and culture

Potato dextrose agar (PDA) was used for the growth of *A. niger* [33]; the spore suspension of *A. niger* was spread on PDA plates and cultured at 35°C for 72 h. Liquefied corn medium was provided by RZBC Co., Ltd. (Rizhao, Shandong, China) and was applied for citric acid fermentation, in which the content of initial total sugar was 184 g L^−1^ and the content of initial reducing sugar was 60.7 g L^−1^. The mineral elements were FeSO_4_ · 7H_2_O 30 mg L^−1^, CaCl_2_ · 2H_2_O 10 mg L^−1^, MnCl_2_ · 4H_2_O 7 mg L^−1^, CuSO_4_ · 5H_2_O 0.3 mg L^−1^, MgSO_4_ · 7H_2_O 0.3 g L^−1^, ZnSO_4_ · 7H_2_O 10 mg L^−1^ and KH_2_PO_4_ 0.3 g L^−1^. The nitrogen resource was 0.004% (w/v) yeast extract. An aliquot of 1 × 10^5^ spore mL^−1^ of *A. niger* was inoculated in liquefied corn medium and cultivated at 0.1 MPa, 330 L h^−1^ ventilatory capacity, 350 r min^−1^ and 35°C for 96 h in 30-L fermentors. All chemicals used were of analytical grade. Antimycin A and 2,4-dinitrophenol (DNP) were purchased from Sigma Chemical, USA. The citric acid test kit was purchased from Boehringer Mannheim Company, FRG. The ATP Colorimetric/Fluorometric Assay Kit was purchased from Biovision Company, USA. The Amplite™ Colorimetric Total NAD^+^ and NADH Assay Kit and the Fluorimetric NADH/NAD^+^ Ratio Assay Kit were purchased from AAT Bioquest Company, USA.

### Determination of citric acid

Approximately 20 mL of fermentation broth at different time points was collected in a 50-mL Eppendorf tube and centrifuged at 10,000 × *g* and 4°C for 5 min. The supernatant was removed in a 10-mL Eppendorf tube and was placed in a water bath at 80°C for 10 min to stop any possible enzymatic reactions; then, the sample was centrifuged again, and the 0.2-mL supernatant was used for the assay. Citric acid was measured enzymatically according to the manufacturer’s instruction (test kit Cat. No. 10139076035). The content of the residual total sugar and residual reducing sugar were determined by DNS (3,5-dinitrosalicylic acid) methods in fermentation broth [34]. All of the assays in this study were performed in triplicate.

### Determination of the growth performance

The amount of 1 × 10^5^ spores mL^−1^ of *A. niger* were inoculated in liquefied corn medium and cultivated at 350 r min^−1^ and 35°C for 72 h, and the diameter of mycelial pellets in the fermentation broth was measured using a Professional Digital Olympus DP7 Microscope (Olympus, Japan). The mycelia were harvested by filtration with suction at different time points and then dried to a constant weight at 60°C for 5 h. The dry cell mass was determined using a Precision Electronic Analytical Balance BS-124S (Shanghai, China).

### Determination of intracellular NADH concentration

The intracellular concentration of NADH was determined using procedures described in previous studies [35,36]. A given amount of the mycelia were rapidly ground into powder with liquid nitrogen, a certain amount of mycelia were quickly sampled into a tube containing 500 μL of ice cold solution (0.4 M KOH for NADH), the samples were immediately mixed with the extractive solution, and the tubes were placed in a water bath at 30°C for 10 min. The KOH extraction (pH 12.3) destroyed the oxidized form of NAD [37]. Then, the tubes were kept on ice for at least 10 min. After neutralization to a pH of 7.4–8.0 using 0.5 M HCl for NADH, the samples were centrifuged at 10,000 × *g*, 4°C for 10 min, and the concentration of NADH in the supernatant was determined using the Amplite™ Colorimetric Total NAD and NADH Assay Kit (AAT Bioquest Product No: 15258). A certain amount of ground powder obtained using the above method was immediately mixed with 500 μL of 0.01 M phosphate buffer (pH 7.4) in a 1.5-mL Eppendorf tube; then the tube were placed in a water-bath at 30°C for 10 min, the samples were centrifuged at 10,000 × *g*, 4°C for 10 min, and the NADH/NAD^+^ levels in the supernatant were measured with the Fluorimetric NADH/NAD^+^ Ratio Assay Kit (AAT Bioquest Product No. 15263) according to the manufacturers’ instructions.

### Determination of intracellular ATP concentrations

Intracellular ATP was extracted using the HClO_4_ method that is widespread for extracting intracellular metabolites [38,39]. Based on the above method, a certain amount of ground powder was sampled quickly into a tube, followed by the addition of 500 μL of 0.6 M HClO_4_, and the powder was mixed by pipette and kept on ice for at least 10 min. The pH was adjusted to 7.2 with 0.1 M KOH. After incubating at 4°C for 30 min, the samples were centrifuged at 10,000 × *g* and 4°C for 10 min. The crystalized KClO_4_ was removed from the solution by filtration (pore size, 0.22 μm), and then the solution was diluted to a volume of 2 mL with 0.01 M phosphate buffer (pH 7.2). The concentration of the intracellular ATP was measured with the ATP Colorimetric/Fluorometric Assay Kit (Product No K354-100) according to the manufacturer’s instruction.

### Statistical analysis

Student’s t-test and p-values were employed to investigate statistical differences, and samples with P <0.05 were considered significant.
